# Thulium fiber laser enucleation of the prostate with SOLTIVE™ SuperPulsed laser systems: a prospective evaluation of early outcomes in an Asian cohort

**DOI:** 10.3389/fruro.2026.1753089

**Published:** 2026-03-30

**Authors:** Brian W.H. Siu, David K.W. Leung, Rachel S.K. Wong, Chris H.M. Wong, Alex Q. Liu, Chi Hang Yee, Jeremy Y.C. Teoh, Peter K.F. Chiu, Chi Fai Ng, Ka Lun Lo

**Affiliations:** 1 S.H. Ho Urology Centre, Department of Surgery, Faculty of Medicine, The Chinese University of Hong Kong, Hong Kong, Hong Kong SAR, China; 2 Li Ka Shing Institute of Health Sciences, The Chinese University of Hong Kong, Hong Kong, Hong Kong SAR, China; 3 Department of Urology, Medical University of Vienna, Vienna, Austria

**Keywords:** BPH, enucleation, soltive, TFL, ThuFLEP, thulium

## Abstract

**Introduction:**

Thulium fiber laser (TFL) technology, with its shorter wavelength and higher water absorption coefficient compared to Holmium: YAG laser, offers theoretical advantages in tissue ablation efficiency and safety profile. We aim to evaluate the early safety and feasibility of Thulium fiber laser enucleation of prostate (ThuFLEP) using the SOLTIVE™ SuperPulsed Laser Systems in the first Asian cohort.

**Methods:**

Thirty-two consecutive men underwent ThuFLEP between October 2022 and September 2025. The primary outcome was the rate of Clavien-Dindo grade ≥3 complications as a safety and feasibility signal, along with assessment of short-to-medium-term functional outcomes. Data are presented as median (interquartile range, IQR). Changes in PSA were analyzed using the Wilcoxon signed-rank test.

**Results:**

The median prostate size was 60 ml (IQR 49.8–76.0 ml), with 50% of patients being catheter dependent. Median operation time was 87 minutes (IQR 73–100 mins). Median enucleated adenoma weight was 31 g (IQR 20–39 g). No Clavien-Dindo grade ≥3 complications occurred. Catheter-free rate was 87.5% on day 1 and 100% by day 14. At 3-month follow-up, median IPSS was 4 (IQR 1–8), median Qmax was 21.5 ml/s (IQR 12.9–28.4 ml/s), with significant PSA reduction (from 6.05 to 1.20 ng/ml; p<0.001). At 12 months (n=25), median Qmax was 21.9 ml/s (IQR 15.2–26.8 ml/s), median IPSS was 2 (IQR 1–5), and there was no surgical or medical retreatment.

**Conclusion:**

ThuFLEP demonstrates early safety and feasibility in this Asian cohort, including catheter-dependent patients. Larger, multicenter studies with longer follow-up are warranted to confirm its safety and efficacy.

**Clinical Trial Registration:**

ClinicalTrials.gov, identifier NCT05292235.

## Introduction

Benign prostatic hyperplasia (BPH) commonly causes lower urinary tract symptoms (LUTS), with 23% risk of acute urinary retention in men aged 60 years (1). While transurethral resection of prostate (TURP) remains the gold standard (2, 3), and minimally invasive surgical therapy (MIST) continues to emerge (4–7), European Association of Urology (EAU) guidelines recommend Holmium laser enucleation of prostate (HoLEP), bipolar enucleation of prostate (BipoLEP), and open prostatectomy for prostates >80 ml (8). Level 1 evidence suggests HoLEP offers favorable perioperative outcomes despite longer operation times compared to TURP (9, 10).

Thulium fiber laser (TFL) has a shorter wavelength than Holmium: YAG (1940 nm vs 2120 nm), closely matching the near-infrared absorption peak of water at 22 °C. The absorption coefficient of TFL is more than four-fold higher than that of Holmium: YAG, resulting in lower threshold and higher ablation efficiency at equivalent pulse energies (11). Lower tissue and water penetration depth may theoretically enhance the safety profile. While these biophysical properties suggest potential advantages, high-quality evidence comparing ThuFLEP with established techniques remains limited (12, 13).

The SOLTIVE™ Premium (Olympus, Tokyo, Japan) TFL is a novel energy source designed for both urolithiasis and prostate enucleation (14). Its wavelength ranges between 1920 and 1960 nm, with energy settings of 0.025–6 J and frequency of 1–2400 Hz. While ThuFLEP series have predominantly been reported in Western populations (12, 15), the correlation between prostate volume and LUTS severity differs between Asian and Caucasian men (16). This study therefore aims to report the early feasibility and safety profile of ThuFLEP using the SOLTIVE™ system in the first prospective Asian cohort.

## Materials and methods

### Study protocol

We established a prospective registry at three centers in Hong Kong: Prince of Wales Hospital, North District Hospital, and Alice Ho Miu Ling Nethersole Hospital. The study protocol was approved by the Joint Chinese University of Hong Kong–New Territories East Cluster Clinical Research Ethics Committee (CRE2022.049). Written informed consent was obtained from all participants, and the study was conducted in accordance with the Declaration of Helsinki. Inclusion criteria were men with indications for BPH surgery (bothersome LUTS, BPH-related bladder stones, haematuria, or acute urinary retention). Exclusion criteria included active urinary tract infection and known functional or structural urinary tract abnormalities (urethral stricture, neurogenic bladder). Clinical trial number: NCT05292235.

After surgery, patients were followed up according to our standardized protocol, including (1) A nurse-led phone consultation scheduled at six weeks, enquiring on symptoms, complications (e.g. haematuria, dysuria, incontinence) and voiding symptom score (e.g. International Prostate Symptoms Score IPSS); (2) A physical follow-up at three months, with questionnaires assessment, uroflowmetry and PSA testing; (3) A follow-up visit at one year. As half of our patients presented with catheter-dependent refractory retention, preoperative IPSS and uroflowmetry were not available for comparison to postoperative data.

The primary outcome was the rate of Clavien-Dindo grade ≥3 complications, serving as an early safety and feasibility signal – consistent with other ThuFLEP series (17). Secondary outcomes included operation time, time to successful trial without catheter (TWOC), enucleation efficiency, morcellation efficiency, PSA change, Qmax, post-void residual (PVR), IPSS, IPSS-QoL, OABSS, and de-novo urinary incontinence (defined as use of ≥1 security pad/day). Uroflowmetry parameters were analyzed only for patients with voided volume ≥125 ml; the questionnaires used in the study (i.e. IPSS and overactive bladder symptom score OABSS) are well validated and utilized in the field of urology (18, 19).

### ThuFLEP procedure

Thulium fiber laser enucleation was performed using the SOLTIVE™ SuperPulsed Laser Systems (Olympus) with a 550 μm laser fiber. The procedure was performed by a single surgeon with over 500 cases of experience in BipoLEP. During enucleation, the setting was 1.5J, 40Hz with short pulse mode; during haemostasis, the setting was 1J, 50Hz with long pulse mode. The two settings were used alternatively to maintain a clear surgical field during enucleation. Both blunt dissection and sharp dissection (with laser) were used. Early apical dissection was performed to protect the external urethral sphincter. Haemostasis was ensured before proceeding with morcellation. The Richard Wolf Piranha morcellator was used (20). During morcellation, two inflow catheters were used to ensure a full bladder. The jaw of the morcellator was ensured to be facing upwards to avoid damaging the trigone and ureteral orifices. Small pieces of prostate adenomas were morcellated within the prostatic fossa at a lower rate to improve efficiency. The other parts of the procedure were performed as previously described in the literature (15, 21). A 24-Fr urethral catheter was inserted postoperatively with bladder irrigation. The first TWOC would be on postoperative day 1. If patients fail TWOC, the next TWOCs would be on day 7 and day 14.

### Statistical analysis

Statistical analyses were performed with SPSS v.26.0 (IBM Corp., Armonk, NY, USA). Continuous variables are presented as median (interquartile range, IQR) and categorical variables as count (percentage). Changes in PSA were assessed using the Wilcoxon signed-rank test. To evaluate the learning curve, patients were divided into three sequential groups - first 10 cases from October 2022 to March 2023, next 10 from April 2023 to November 2023, last 12 November 2023 to September 2025 - and enucleation and morcellation efficiencies were compared using the Kruskal–Wallis test. A two-sided p-value<0.05 was considered significant.

## Results

### Patient characteristics and perioperative outcomes

Thirty-two consecutive men underwent ThuFLEP between October 2022 and September 2025. Baseline characteristics and perioperative outcomes are summarized in **Table 1**. Median age was 70.5 years (IQR 67.0–75.0 years). Median prostate size was 60.0 ml (IQR 49.8–76.0 ml). Indications for surgery were acute urinary retention (including refractory retention) (65.6%), BPH-related bladder stones (15.6%), haematuria (9.4%), and bothersome LUTS (9.4%). Overall, half of the patients (16/32) were catheter-dependent preoperatively.

**Table 1:** Patient characteristics and perioperative outcomes (n=32).

Characteristic	Value
Demographics	
Median age at operation (years)	70.5 (67.0–75.0)
Median preoperative prostate size (ml)	60.0 (49.8–76.0)
Preoperative PSA (ng/ml)	6.05 (3.67–11.25)
ASA grading	2 (2–2)
Indication	
Acute urinary retention (including refractory retention)	21 (65.6%)
BPH-related bladder stones	5 (15.6%)
BPH-related haematuria	3 (9.4%)
Bothersome LUTS	3 (9.4%)
Preoperative catheter dependence	16 (50%)
Perioperative outcomes	
Operation time (min)	87 (73–100)
Enucleation time (min)	30 (20–40)
Morcellation time (min)	30 (21–40)
Enucleated adenoma weight (g)	31 (20–39)
Enucleation efficiency (g/min)	0.98 (0.60–1.54)
Morcellation efficiency (g/min)	0.93 (0.70–1.24)
Duration of bladder irrigation (hours)	6 (6–6)
Catheter-free rate by day 1	28 (87.5%)
Catheter-free rate by day 7	31 (96.9%)
Catheter-free rate by day 14	32 (100%)
Postoperative Hb drop (g/dl)	1.2 (0.5–1.8)
Transfusion requirement	1 (3.1%)
30-day complications	
Clavien-Dindo grade 1–2	4 (12.5%)
– Acute urinary retention	2 (6.3%)
– Epididymo-orchitis	1 (3.1%)
– Haematuria requiring transfusion	1 (3.1%)
Clavien-Dindo grade ≥3	0
Length of stay (hours)	28 (26–30)
6-week outcomes (n=32)	
IPSS	6 (3–10)
IPSS-QoL	2.5 (2–3)
OABSS	3 (2–6)
*3-month outcomes (n=32)*	
PSA (ng/ml)	1.20 (0.40–1.79)
PSA reduction (%)	75.8% (p<0.001)*
Voided volume (ml)	177.0 (140.6–299.5)
Patients with voided volume ≥125 ml	25 (78.1%)
Qmax (ml/s)†	21.5 (12.9–28.4)
PVR (ml)†	50 (26–66)
IPSS	4 (1–8)
IPSS-QoL	2 (2–3)
OABSS	3 (1–5)
Urinary incontinence	1 (3.1%)
12-month outcomes (n=25)	
Voided volume (ml)	277.2 (188.2–330.5)
Patients with voided volume ≥125 ml	18 (72%)
Qmax (ml/s)†	21.9 (15.2–26.8)
PVR (ml)†	41 (32–65)
IPSS	2 (1–5)
IPSS-QoL	1 (1–2)
OABSS	1 (1–1)
Urinary incontinence	0
Surgical retreatment rate	0
Use of alpha-blockers or 5-ARIs	0

Data are presented as median (IQR) or n (%).

*Wilcoxon signed-rank test.

†Patients with voided volume <125 ml were excluded from Qmax and PVR analysis.

PSA, prostate-specific antigen; ASA, American Society of Anesthesiologists; LUTS, lower urinary tract symptoms; IPSS, International Prostate Symptom Score; QoL, quality of life; OABSS, Overactive Bladder Symptom Score; PVR, post-void residual; 5-ARI, 5-alpha reductase inhibitors.

No Clavien-Dindo grade ≥3 complications occurred postoperatively. Four grade 1–2 complications (12.5%) were observed: two cases of recurrent urinary retention (managed with re-catheterization and successful TWOC by day 7), one epididymo-orchitis (resolved with antibiotics), and one haematuria requiring blood transfusion. Median operation time was 87 minutes (IQR 73–100 mins). Median enucleated adenoma weight was 31 g (IQR 20–39 g). Enucleation efficiency was 0.98 g/min (IQR 0.60–1.54 g/min), and morcellation efficiency was 0.93 g/min (IQR 0.70–1.24 g/min). Successful TWOC on day 1 was achieved in 87.5%; by day 7, 96.9% were catheter-free, and all were catheter-free by day 14. Median postoperative hemoglobin drop was 1.2 g/dl (IQR 0.5–1.8 g/dl). Median length of stay was 28 hours (IQR 26–30 hours).

### Postoperative outcomes

All 32 patients completed 3-month follow-up. (**Table 1**) At 3 months, median IPSS was 4 (IQR 1–8), and median IPSS-QoL was 2 (IQR 2–3). Of these, 25 patients had voided volume ≥125 ml: median Qmax was 21.5 ml/s (IQR 12.9–28.4 ml/s), median PVR was 50 ml (IQR 26–66 ml). PSA decreased significantly from a median of 6.05 ng/ml (IQR 3.67–11.25 ng/ml) preoperatively to 1.20 ng/ml (IQR 0.40–1.79) at 3 months (median reduction 75.8%; p<0.001 by the Wilcoxon signed-rank test). One patient (3.1%) reported stress urinary incontinence (requiring two pads/day) at 3 months; he was prescribed pelvic floor exercises and was continent at 12 months.

Twenty-five (78.1%) patients completed twelve months of follow-up, one (3.1%) patient died at seven months from chest infection, four (12.5%) patients defaulted follow-up, and two (6.3%) patients had not yet reached the follow-up timepoint. The median IPSS was 2 (IQR 1–5), and median IPSS-QoL was 1 (IQR 1–2). Of these, 18 patients had voided volume ≥125 ml: median Qmax was 21.9 ml/s (IQR 15.2–26.8 ml/s), median PVR 41 ml (IQR 32–65 ml). No patient required surgical retreatment or alpha-blockers or 5-alpha reductase inhibitors at follow-up. There was no urinary incontinence.

### Learning curve analysis

To assess the impact of the learning curve, patients were divided chronologically into three groups: first 10 cases from October 2022 to March 2023, next 10 from April 2023 to November 2023, last 12 November 2023 to September 2025. In Group 1, the median enucleation efficiency was 0.86 g/min (IQR 0.56–1.62), and the median morcellation efficiency was 0.98 g/min (IQR 0.63–1.39). For Group 2, the median enucleation efficiency was 0.98 g/min (IQR 0.73–1.16), with a median morcellation efficiency of 0.93 g/min (IQR 0.63–1.18). In Group 3, the median enucleation efficiency was 1.00 g/min (IQR 0.59–1.63), and the median morcellation efficiency was 0.90 g/min (IQR 0.75–1.58). The Kruskal–Wallis test showed no significant difference across groups for enucleation efficiency (p=0.973) or morcellation efficiency (p=0.785). (**Table 2**).

**Table 2:** Enucleation and morcellation efficiency across sequential case groups.

Parameter	Group 1 (cases 1–10)	Group 2 (cases 11–20)	Group 3 (cases 21–32)	p-value
Enucleation efficiency (g/min)	0.86 (0.56–1.62)	0.98 (0.73–1.16)	1.00 (0.59–1.63)	0.973
Morcellation efficiency (g/min)	0.98 (0.63–1.39)	0.93 (0.63–1.18)	0.90 (0.75–1.58)	0.785

Data are presented as median (interquartile range).

## Discussion

This prospective study demonstrates the feasibility and early safety of ThuFLEP using the SOLTIVE™ system in an Asian population, with a substantial proportion of catheter-dependent patients.

When contextualizing our results within the current literature, several comparisons are informative. Our median 3-month Qmax of 21.5 ml/s and IPSS of 4 are consistent with those reported in the literature. In the prospective randomized trial by Kosiba et al. comparing ThuFLEP with HoLEP in 150 patients, no statistically significant differences were observed between the two techniques. At three months, median IPSS was 8.5 in the ThuFLEP group versus 7 in the HoLEP group (p > 0.9), and median Qmax was 19 ml/s versus 17 ml/s respectively (p > 0.9) (21). In the randomized trial by Bozzini et al. comparing thulium:YAG laser enucleation (ThuLEP) with continuous-wave thulium fiber laser enucleation (CW-ThuFLEP) in 110 patients, the 3-month Qmax was 26.5 ml/s versus 27.1 ml/s, and the 3-month IPSS was 5.1 versus 5.8, respectively. The authors concluded that theoretical advantages of TFL did not translate into clinically relevant differences in perioperative outcomes (12). Our observed enucleation efficiency of 0.98 g/min was also consistent with Bozzini et al. (0.79 g/min). The low incontinence rate in our study (3.1% transient stress incontinence) could be attributed to our emphasis on early apical dissection and sphincter preservation.

A systematic review and meta-analysis by Uleri et al. synthesized evidence from five studies including 1287 HoLEP and 1555 ThuFLEP patients (15). They found that ThuFLEP was associated with a better IPSS at 3 months, though the difference was not clinically meaningful (mean difference 0.59, p < 0.001). No difference was found for IPSS at 6–12 months (p = 0.9) or for IPSS-QoL at any timepoint. HoLEP was associated with a better Qmax at 3 months (mean difference 1.41 ml/s, p = 0.002) and ThuFLEP at 6–12 months (mean difference -2.61 ml/s, p = 0.01), but again these differences were not clinically significant. No difference was found in major or overall complication rates, though HoLEP was associated with shorter enucleation time (mean difference -11.86 minutes, p = 0.03). In a propensity-score matched analysis from the REAP registry, Gauhar et al. reported higher Qmax and lower PVR with ThuFLEP versus HoLEP at 1 year (13), while randomized trials have shown equivalent outcomes (22, 23).

A recent single-surgeon series from Belgium demonstrated that the learning curve of ThuFLEP was nearly 60 cases, yet the complication rates remained low throughout the training process (24). With just over 30 cases of experience, there remains a learning curve towards the end of our series. Similarly, we have grouped the cases in our series chronologically to illustrate the learning curve experience. While we corroborate with the Belgian series in the low complication rate during training process, the lack of significant differences in efficiency across the three sequential groups could be due to sample size.

Strengths of this study include the prospective design and standardized follow-up. Moreover, amongst the growing body of ThuFLEP evidence, it is important to report Asian experiences due to difference in LUTS and prostate characteristics (16). The inclusion of 50 percent of catheter-dependent patients reflects the real-world population, suggesting ThuFLEP’s feasibility and early safety using the SOLTIVE™ system.

Limitations include the single-arm, single-surgeon design and modest sample size (n=32), which limits generalizability and power to detect complications. The lack of a control group precludes comparative effectiveness claims; longer follow-up is needed to assess durability. Future research should focus on head-to-head comparisons with established techniques (HoLEP, TURP, BipoLEP) with longer follow-up duration.

## Conclusions

ThuFLEP with the SOLTIVE™ system demonstrates early safety and feasibility in this Asian cohort, including catheter-dependent patients. Larger, multicenter studies with longer follow-up are warranted to confirm its safety and efficacy.

## Data Availability

The raw data supporting the conclusions of this article will be made available by the authors, without undue reservation.
